# Nonmonotonic contactless manipulation of binary droplets via sensing of localized vapor sources on pristine substrates

**DOI:** 10.1126/sciadv.aba3636

**Published:** 2020-09-30

**Authors:** Robert Malinowski, Ivan P. Parkin, Giorgio Volpe

**Affiliations:** Department of Chemistry, University College London, 20 Gordon Street, London WC1H 0AJ, UK.

## Abstract

Droplet motion on surfaces influences phenomena as diverse as microfluidic liquid handling, printing technology, and energy harvesting. Typically, droplets are set in motion by inducing energy gradients on a substrate or flow on their free surface. Current configurations for controllable droplet manipulation have limited applicability as they rely on carefully tailored wettability gradients and/or bespoke substrates. Here, we demonstrate the nonmonotonic contactless long-range manipulation of binary droplets on pristine substrates due to the sensing of localized water vapor sources. The droplet-source system presents an unexpected off-centered equilibrium position. We capture the underlying mechanism behind this symmetry breaking with a simplified model based on the full two-dimensional functional form of the surface tension gradient induced by the source on the droplet’s free surface. This insight on the transport mechanism enables us to demonstrate its versatility for applications by printing, aligning, and reacting materials controllably in space and time on pristine substrates.

## Teaser

Remote localized vapor sources manipulate binary droplets in a nonmonotonic way on pristine surfaces for various applications.

## INTRODUCTION

Droplets moving on solid surfaces are at the heart of many phenomena of fundamental and applied interest in physics, biophysics, chemistry, and materials science (*1*, *2*). Examples include “tears of wine” due to the Marangoni effect (*3*), rolling droplets on self-cleaning substrates (*4*), microfluidic liquid handling (*5*), as well as enhancing heat transfer (*6*), sensing (*7*), and printing technologies (*8*). Achieving real-time control over the directionality of moving droplets is therefore an important milestone toward harnessing them for applications in, e.g., printable materials (*8*), biological assays (*9*), and microreactors (*10*).

The main impediment to trigger droplet motion on solid surfaces comes from the hysteresis of the contact angle that pins the droplet’s edge to the underlying substrate (*11*). The generation of gradients of surface energy on the substrate is a widespread strategy to overcome the pinning of the contact line and achieve motion (*5*, *6*, *12*–*14*). Alternatively, thermal and solutal imbalances of surface tension can be directly induced on the droplet’s free surface, thus inducing flows within the droplet that ultimately lead to its motion (*7*, *15*, *16*). For example, vapor fields can be used to induce such imbalances and influence droplet physics and motion (*7*, *17*–*21*). In an emblematic recent example, multiple aqueous droplets of food coloring [containing different concentrations of propylene glycol (PG) in water] showed a wealth of emerging spontaneous behaviors when placed on a glass substrate, including attraction, chasing, coalescence, and repulsion (*7*). By modifying the underlying substrate or by developing elaborate vapor traps with multiple droplets, various examples of droplet-based devices were also demonstrated (*7*).

Because of their widespread use in technology and their innate ability to overcome contact angle hysteresis (*8*, *18*), similar droplets of two (or more) liquid components of different volatilities are therefore prime candidates for applications that require control over droplet motion on surfaces. When it comes to controlling the directional transport of droplets (i.e., manipulating them), however, existing techniques rely on large gradients of surface energy (*6*, *12*), on carefully engineered substrates (*5*, *14*), or on tailored trapping potentials (*7*), thus limiting the level of control that one can achieve over droplet motion and its applicability on pristine substrates, for example, for printing technology.

Here, we demonstrate the nonmonotonic contactless long-range two-dimensional (2D) manipulation of binary droplets of water and PG on solid pristine surfaces. These droplets spontaneously move in a directional manner in response to small imbalances of surface tension gradients induced by the presence of an external localized source of water vapor. Unexpectedly, unlike previous observations (*7*), a droplet of fixed initial composition can move either away or toward a vapor source of fixed composition depending on separation distance. We show with a simplified analytical model based on the 2D functional form of the surface tension gradient along the droplet’s free surface that the viscous nature of the driving forces at play (<μN) is paramount to capture the observed dual response (from attractive to repulsive) of the droplet with distance from the source. Our robust understanding of the underlying motion mechanism allows us to showcase its adaptability in a range of potential applications in materials science, including pattern formation, the printing of chemical gradients, and the mixing of reactive materials on pristine substrates (i.e., without requiring further modification of the underlying surface).

## RESULTS

### Motion of binary droplets under an external vapor source

**Fig. 1:**
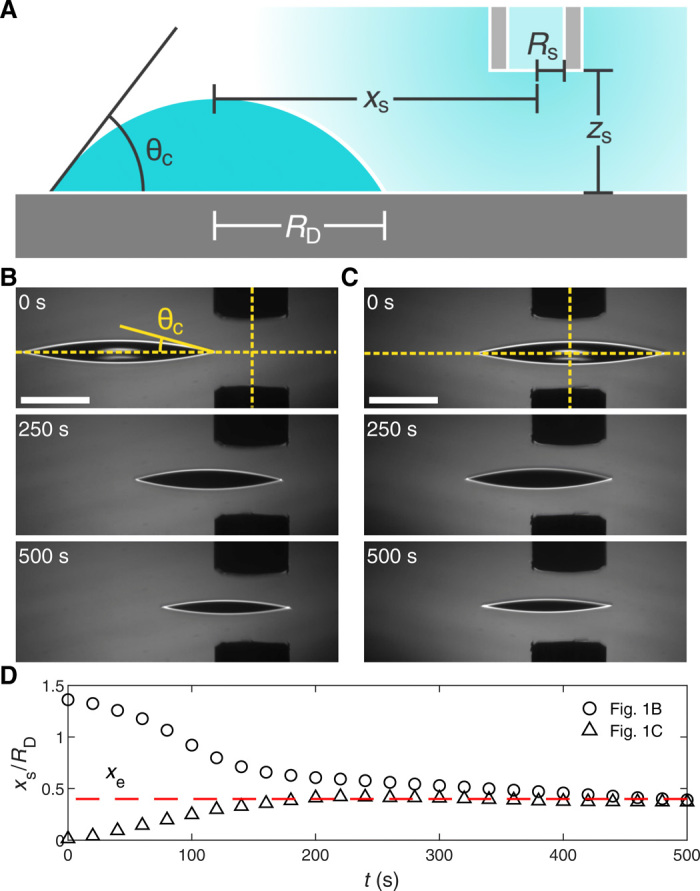
Binary droplets under an external vapor source: attraction versus repulsion. (**A**) Schematics of the contactless manipulation of binary droplets on solid substrates with an external localized vapor source: We placed a binary droplet (radius *R*_D_; contact angle θ_c_) at a distance *x*_s_ from a blunt needle (inner radius *R*_s_) from where water vapor diffuses; *z*_s_ is the distance between source and substrate. We performed all experiments within an environmental chamber with controlled temperature (*T* = 21 ± 0.5 ° C) and relative humidity (*RH* = 50 ± 5%). (**B** and **C**) Time sequences showing a 0.5-μl binary droplet of water and PG (*R*_D_ = 1.38 ± 0.06 mm; θ_c_ = 12.5 ± 0. 7^∘^; mole fraction of water *x*_H_2_O_ = 0.95) being (B) attracted to or (C) repelled from the source (*R*_s_ = 350 μm) depending on the initial separation *x*_s_: (B) *x*_s_ = 2 mm (movie S1) and (C) *x*_s_ = 0 (movie S2). The horizontal and vertical dashed lines highlight the substrate and the center of the vapor source, respectively. Scale bars, 1 mm. (**D**) Time evolution of *x*_s_ converging to the same radial distance *x*_e_ (dashed line) from the source for the droplets in (B) (circles) and (C) (triangles).

In the most basic configuration, we deposited a 0.5-μl binary droplet of water and PG (radius *R*_D_ = 1.38 ± 0.06 mm; mole fraction of water *x*_H_2_O_ = 0.95) on a clean glass slide at a distance *x*_s_ from an external localized source of water vapor (Fig. 1A) (see Materials and Methods). As evaporation is faster at the droplet’s edges (*22*) and PG is less volatile and with a lower surface tension than water, a radially symmetric gradient of surface tension γ forms on the droplet’s free surface (*7*, *23*). The resulting inward Marangoni stresses prevent spreading (*24*) so that, due to its ongoing evaporation, the droplet features a contact angle (θ_c_ = 12.5 ± 0. 7^∘^) higher than either of the pure liquids and a reduced hysteresis (*23*). When *x*_s_ → ∞ (i.e., in the absence of the source), the two-component droplet with a spherical cap shape is stationary on a flat surface; however, in the presence of the source, because of the reduced hysteresis, the binary droplet can instead move, maintaining a spherical cap shape if the contact line is not pinned as in our case (Fig. 1 and fig. S1). In particular, when placed afar (*x*_s_ = 2 mm; Fig. 1B and movie S1), the droplet experiences an attractive force toward the source. This attractive behavior is consistent with a relative larger local increase in humidity at the droplet’s edge nearest to the source, which generates motion by slowing down evaporation and comparatively increasing the value of surface tension at that edge with respect to the opposite side, similar to previous observations (*7*, *25*). Such considerations based on differences of absolute values of surface tension would naturally lead one to expect a stable equilibrium position to appear directly beneath the source’s center (*x*_s_ = 0), as any displacement from *x*_s_ = 0 would induce a restoring force. Instead, *x*_s_ = 0 is an unstable equilibrium position from which the droplet gets easily repelled to an off-centered radial distance *x*_e_ (Fig. 1C and movie S2), where the droplet consistently settles near the end of the evaporation whether it starts from afar the vapor source or below it (Fig. 1D). This dual response (i.e., from attractive to repulsive) of the droplet motion with distance from the source is fundamentally different from that observed in previous reports (*7*), where a binary droplet of water and PG with our initial composition always showed attraction to a pure source of water vapor.

### Dynamics of droplet’s motion

**Fig. 2:**
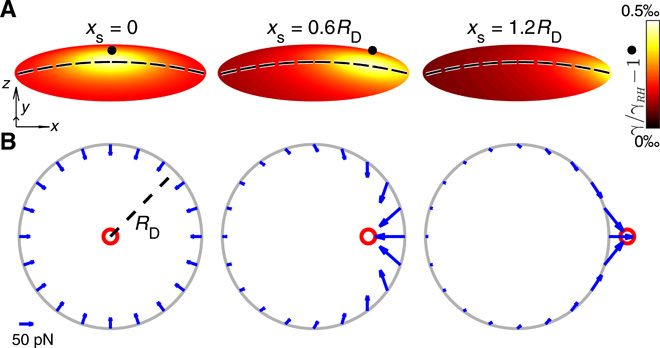
Forces induced on the contact line of binary droplets under an external vapor source. An external vapor source induces gradients of surface tension γ on a binary droplet’s free surface. The corresponding Marangoni flows generate forces on the droplet’s contact line, ultimately leading to its motion toward or away the vapor source. (**A**) Estimated change of surface tension γ along the droplet’s free surface due to vapor sources (black dots, *R*_s_ = 350 μm) placed directly above (*x*_s_ = 0), with a slight offset (*x*_s_ = 0.6*R*_D_) and afar from (*x*_s_ = 1.2*R*_D_) the droplet when evaporation starts. The change in γ is given relative to a reference value γ*_RH_* estimated removing the effect of the source in Eq. 2, i.e., by imposing *p*_H_2_O_ = *p_RH_*. Dashed lines, meridians through the droplets’ apices. The coordinate unit vectors correspond to 0.5 mm. (**B**) Calculated local force (blue arrows) along the contact line (gray circles) due to vapor sources as in (A) (red circles). Force vectors, shown every $\frac{\pi }{10}$, are integrated over $\frac{\pi }{500}$ intervals along the contact line.

To efficiently harness this mechanism, we have developed a simplified analytical model to better understand our counterintuitive experimental observations. This duality (attraction versus repulsion) in the interaction between droplet and source cannot be simply explained by arguments purely based on differences of absolute values of surface tension on opposing sides of the droplet or between its edge and an adjacent precursor film, for which the droplet would always move toward the vapor source (*7*, *25*). Instead, as can be seen in Fig. 2, both the magnitude and directionality of the net force experienced by the droplet can be modulated when we account for the full 2D functional form of the surface tension gradient along the droplet’s free surface. Our analytical derivation relies on the initial estimation of the droplet’s composition and composition-dependent physical parameters from our experimental data (see Materials and Methods).

To a first approximation, we can understand the whole process assuming a steady state for the diffusion of water vapor from the source toward the droplet’s free surface. In the presence of the vapor source, aqueous vapor saturates the atmosphere within a blunt needle [vapor pressure *p*_s_ = 2.49 kPa at *T* = 21 ° C (*26*)] and diffuses toward the droplet’s free surface, where it induces a local decrease in the evaporation rate proportional to the local water partial pressure, *p*_H_2_O_(*r*, θ, *h*), with *r*, θ, and *h*(*r*) being variables describing the free surface in cylindrical coordinates (fig. S2A). Considering the source as a flat circular disc of radius *R*_s_ at pressure *p*_s_ and centered in (*x*_s_, *y*_s_, *z*_s_) (fig. S2A), neglecting any influence from the droplet on the diffusion from it and assuming the vapor to behave ideally, we can calculate *p*_H_2_O_(*r*, θ, *h*) as the sum of two terms (see derivation S1)

$$p_{H_{2}O}(r,\theta ,h)=P_{H_{2}O}(r,\theta ,h)+{P\prime }_{H_{2}O}(r,\theta ,h)$$ (1)

where $P_{H_{2}O}(r,\theta ,h)=\frac{2(p_{s}-p_{\mathrm{RH}})}{\pi }\text{sin}-1(\frac{2R_{s}}{\sqrt{{\left(d-R_{s}\right)}^{2}+{\left(z_{s}-h\right)}^{2}}+\sqrt{{\left(d+R_{s}\right)}^{2}+{\left(z_{s}-h\right)}^{2}}})+p_{\mathrm{RH}}$ is the steady-state solution to the diffusion problem of water vapor from the disc source (*27*) at ambient relative humidity *RH* (eq. S1), and ${P\prime }_{H_{2}O}(r,\theta ,h)=\frac{2(p_{s}-p_{\mathrm{RH}})}{\pi }\text{sin}-1(\frac{2R_{s}}{\sqrt{{\left(d-R_{s}\right)}^{2}+{\left(z_{s}+h\right)}^{2}}+\sqrt{{\left(d+R_{s}\right)}^{2}+{\left(z_{s}+h\right)}^{2}}})$ is a correction term introduced to account for the presence of the substrate (eq. S2) (*28*). Here, $d(r,\theta )=\sqrt{{\left(x_{s}-r\text{cos}\;\theta \right)}^{2}+{\left(y_{s}-r\text{sin}\;\theta \right)}^{2}}$, $h(r)=\frac{\left(R_{D}^{2}-r^{2}\right)}{2R_{D}}\theta_{c}$ (*29*), and *p_RH_* is the partial pressure of water corresponding to *RH*. For simplicity, we have assumed that the presence of the droplet does not alter the solution of the diffusion problem from the source. This approximation becomes increasingly valid at higher droplet-source separation distances, as the full coupling among the external vapor phase and the droplet’s composition can be quite complex otherwise (*30*). Nonetheless, as we will see below, there is good agreement between our model and our experimental data.

The local decrease in evaporation rate due to *p*_H_2_O_(*r*, θ, *h*) induces local variations in composition χ_H_2_O_(*r*, θ, *h*), and hence in local surface tension γ(*r*, θ, *h*), along the droplet’s free surface (see derivation S2). Given χ_H_2_O_(*r*, θ, *h*), we can estimate γ(*r*, θ, *h*) with empirical formulae (*31*) and use it to estimate the gradient in surface tension along the free surface (fig. S3) to determine the forces acting on the droplet’s contact line (Fig. 2). Neglecting intrinsic imbalances of composition due to the evaporation of the binary droplet (*24*) and assuming that the composition perturbations due to the source are small, we can thus express the local water composition along the free surface as the local mole fraction

$$\chi_{H_{2}O}(r,\theta ,h)=\frac{x_{H_{2}O}^{\tau }V_{m}^{-1}(x_{H_{2}O}^{\tau })+{\mathrm{kp}}_{H_{2}O}}{V_{m}^{-1}(x_{H_{2}O}^{\tau })+{\mathrm{kp}}_{H_{2}O}}$$ (2)

where $x_{H_{2}O}^{\tau }(t)$ is a reference value for the time-varying bulk composition of the evaporating droplet (see derivations S2 and S3), $V_{m}(x_{H_{2}O}^{\tau })$ is the molar volume of the mixture at $x_{H_{2}O}^{\tau }$ as estimated from empirical formulae (*32*), and *kp*_H_2_O_ is the additional amount of water retained along the droplet’s free surface due to the slower local evaporation that we assume proportional to *p*_H_2_O_ (see derivation S2). The proportionality constant *k* expresses the balance of the rate constants associated to the condensation and vaporization of the additional amount of water along the free surface when we assume an equilibrium between the two processes.

As a consequence of these local changes in composition, gradients of surface tension form over the droplet’s free surface (fig. S3) that drive the formation of Marangoni flows toward the areas of higher γ (*33*), i.e., where the evaporation is slower. The estimated values in Fig. 2A show how the position of this maximum shifts along the droplet’s free surface following the position of the source, moving from the droplet’s apex for *x*_s_ = 0 to its contact line when the source is far away (*x*_s_ = 1.2*R*_D_). For no displacement (*x*_s_ = 0), the gradient in surface tension, and hence the corresponding Marangoni flows along the free surface, is radially symmetric and pointing inward toward the droplet’s apex (fig. S3). When we displace the source toward one edge, the flows in the droplet become radially asymmetric (*34*), strengthening under the source at first due to a steeper gradient in surface tension (fig. S3). When the source is far away, the flows coming from the distal edge predominate instead due to the confined geometry of the droplet. Between these two cases, the gradient in surface tension at the edge closer to the source changes directionality (fig. S3). Although the coupling between droplet composition, internal flows, and external vapor phase is rather simplified in our derivation (*30*), this change in surface tension gradient intuitively justifies the dual response of the droplet’s motion to the position of the source (Fig. 1).

To formalize this intuition, we can integrate the viscous stress induced by the flows within the droplet on the liquid-solid interface to obtain a viscous driving force $F_{x}^{\gamma }$ in the direction *x* of motion. For a droplet of small constant contact angle θ_c_ that maintains a spherical cap shape while moving at constant velocity *v_x_* along *x*, the liquid flow within the droplet, in the lubrication approximation, is the superposition of a Poiseuille flow induced by the pressure gradient in the moving droplet and a Marangoni flow due to the gradient in surface tension at the droplet’s free surface (*35*). Assuming no deformation of the droplet and small gradients, the viscous stress on the substrate in the direction of motion due to these flows is given by (*35*)

$$\sigma_{\mathrm{xz}}(z=0)=3\eta \frac{u_{x}(x,y)}{h(x,y)}-\frac{1}{2}\frac{\partial \gamma (x,y)}{\partial x}$$ (3)

where η is the dynamic viscosity of the mixture estimated with empirical formulae at the droplet’s composition (*32*), and *u_x_* is the projection of the local velocity of the contact line in the *x* direction. By integrating σ*_xz_*(*z* = 0) over the droplet’s basal area in polar coordinates, we can calculate the total viscous force in the *x* direction as the sum of two terms, $F_{x}^{v}=F_{x}^{u}-F_{x}^{\gamma }$ (see derivation S4), using the fact that dissipation (i.e., the viscous force) is dominated by the edge where the shear gradient is the sharpest (*35*). We thus obtain that the viscous drag force on the moving droplet is given by

$$F_{x}^{u}\approx \frac{3\pi \eta R_{D}\ell_{n}v_{x}}{\theta_{c}}$$ (4)

where ℓ*_n_* = 11.2 is a constant prefactor determined by the droplet’s geometry and the molecular dimension of the liquid (*7*, *35*). The viscous driving force is instead given by

$$F_{x}^{\gamma }=\int_{0}^{2\pi }\frac{R_{D}}{2}(R_{D}\frac{\partial \gamma }{\partial r}\text{cos}\;\theta -\frac{\partial \gamma }{\partial \theta }\text{sin}\;\theta )\;d\theta$$ (5)

**Fig. 3:**
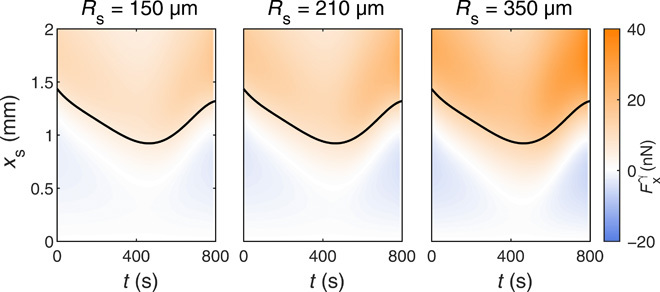
Calculated net viscous driving force exerted on binary droplets by an external vapor source. Calculated viscous driving force $F_{x}^{\gamma }$ (Eq. 5) exerted on the droplet by the vapor source as a function of the distance *x*_s_ from the source and time *t* from the beginning of the evaporation for increasing source radii *R*_s_. The solid lines represent the time evolution of the droplet’s radius *R*_D_ (fig. S2B).

In the absence of surface wettability gradients or external forces acting on the droplet (as in our case), the balance of forces requires that $F_{x}^{v}=0$ so that $F_{x}^{u}=F_{x}^{\gamma }$. Because of the symmetry in the problem, the net viscous force along the *y* direction is null instead. Our transport mechanism is entirely driven and dominated by viscous forces. Figure 2B shows how the integrated function in Eq. 5 (with units of force) varies along the contact line for three different source positions at the start of the evaporation. This quantity always points toward the source and intensifies in the parts of the contact line closer to it. These two considerations alone can formally explain our main observations in Fig. 1. When the source is just above the droplet’s apex (*x*_s_ = 0), there is no net force because of symmetry; this is, however, an unstable configuration as any small displacement generates repulsion (*x*_s_ = 0.6*R*_D_). When the source crosses the contact line (*x*_s_ = 1.2*R*_D_), the droplet starts experiencing a net attractive force instead, and a stable equilibrium point is formed at the crossover between attraction to and repulsion from the source. Overall, this force primarily acts on the contact line, is a function of the gradient of surface tension, and has typical values <μN consistent with previous reports (Fig. 3) (*7*). Last, by solving the force balance equation for the moving droplet, we can calculate its velocity *v_x_* in the vapor field generated by the source as

$$v_{x}=\frac{\theta_{c}}{6\pi \eta \ell_{n}}\int_{0}^{2\pi }(R_{D}\frac{\partial \gamma }{\partial r}\text{cos}\;\theta -\frac{\partial \gamma }{\partial \theta }\text{sin}\;\theta )\;d\theta$$ (6)

**Fig. 4:**
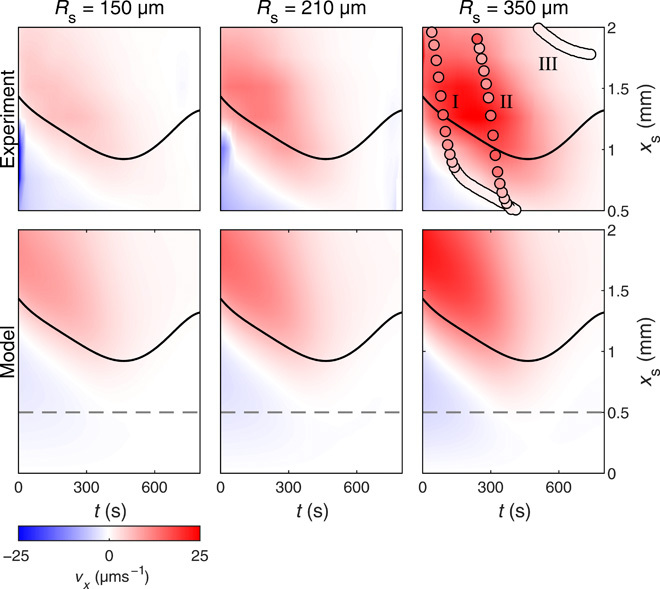
Droplet’s velocity in experiments and model. Droplet’s mean velocity *v*_*x*_ with distance *x*_s_ from the source and time *t* from when evaporation starts for increasing *R*_s_ (*R*_s_ = 150 μm, *R*_s_ = 210 μm, and *R*_s_ = 350 μm) in experiments and simulations. Mean values are averages of at least five different experiments with a SD of 0.5 μm s^−1^ at *R*_s_ = 150 μm, 0.8 μm s^−1^ at *R*_s_ = 210 μm, and 1.5 μm s^−1^ at *R*_s_ = 350 μm. We performed these experiments by continuously moving the stage to keep the distance between source and droplet fix at an initial set value *x*_s_ (see Materials and Methods). The solid lines represent the time evolution of the droplet’s radius *R*_D_ (fig. S2B). In the model values, the dashed lines delimit the part where corresponding experiments are available. In the experiments for *R*_s_ = 350 μm, the circles represent three trajectories of individual droplets moving toward the source from *x*_s_ = 2 mm (as in Fig. 1B) at different times (fig. S4): (I) 30 s, (II) 250 s, and (III) 500 s from the beginning of their evaporation. The color code of each circle represents the droplet’s velocity at a given time.

Typical experimental values of *v_x_* range between a few and a few tens of μm s^−1^, and are well reproduced by our model (Fig. 4). Throughout our derivation, we have assumed that the droplet maintains the same spherical cap shape when attracted toward the source or repelled from it. This is a valid assumption even for the biggest vapor source considered here (fig. S1). Nonnegligible deformations might, however, take place in the presence of even stronger vapor fields (*34*).

As the droplet’s geometry and composition are changing due to evaporation (fig. S2 and derivation S3), both force and velocity vary in time too (Figs. 3 and 4). The experiments in Fig. 4 show how the droplet’s velocity evolves in time and with the distance *x*_s_ from the source as a function of the source size *R*_s_. Qualitatively, these maps show the same common features, which are well reproduced by our model. At the beginning of the evaporation process, a small region of null speed and force (white, Figs. 3 and 4) in the proximity of the droplet’s edge separates a repulsive area (blue) from an attractive one (red). As the droplet’s radius is initially shrinking (fig. S2B), the stable equilibrium point of null speed, and hence the limit of the repulsive zone, shifts toward the droplet’s center with time, giving way to the attractive zone. When moving away from the droplet’s edge, this attractive region presents a time-dependent maximum around 〈*x*_s_〉 ≈ 1.3*R*_D_ as the strength of the attractive force fades when the distance from the source increases due to the weaker vapor fields around the droplet. Although the force strengthens toward the end of the evaporation (Fig. 3), speed drops eventually to zero as the droplet becomes richer in PG over time (and its viscosity increases) so that substantially higher forces are needed to set it in motion. As can be seen in the simulated maps (Figs. 3 and 4), a region of null speed and force emerges also for *x*_s_ ≈ 0. As previously observed (Fig. 1), this represents an unstable equilibrium point, which is surrounded by a region of repulsive forces (Fig. 3). We can attribute all qualitative differences (also reproduced by our model) to the source size, i.e., the strength of its influence at a given *x*_s_ (Fig. 4). In particular, larger sources exert larger attractive forces (Fig. 3) and, as a consequence, droplets move faster and experience the source influence from further away and for longer during their evaporation. To keep a fixed distance *x*_s_ between droplet and source over time, we performed these experiments by continuously moving the microscope stage to compensate for the droplet’s motion (see Materials and Methods). When droplets are free to move on the substrate as in Fig. 1, they follow trajectories on the maps in Fig. 4 with a time-varying velocity consistent with the underlying functional form of these velocity surfaces (fig. S4) until they settle in the stable region of null speed.

### Applications of moving droplets under an external vapor source

**Fig. 5:**
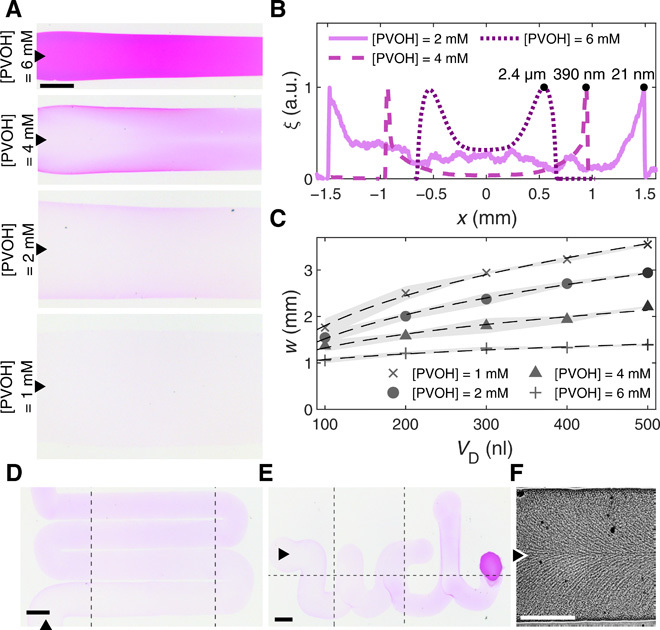
Printing with moving droplets. (**A**) Photographs of linear polymer deposits (PVOH) from moving water/PG droplets (*V*_D_ = 0.5 μl, *x*_H_2_O_ = 0.95) with decreasing PVOH concentrations. (**B**) Typical height profiles for the deposits in (A) (see Materials and Methods). a.u., arbitrary units. (**C**) Deposit maximum line width *w* as a function of droplet’s volume and PVOH concentration, averaged across at least three deposits. The dashed lines are fits to the experimental data showing the proportionality between *w* and $V_{D}^{1/3}$. (**D** and **E**) Stitched photographs of PVOH deposits ([PVOH] = 2 mM) from moving water/PG droplets (*V*_D_ = 0.1 μl, *x*_H_2_O_ = 0.95) guided along 2D patterns to form (D) a serpentine with an ≈30-μm interline spacing (movie S3) and (E) the letters ucl (movie S4). (**F**) Example photograph (enhanced with an edge-aware filter for contrast) of alignment in linear polymer deposits ([PEG] = 100 mM) from moving water/PG droplets (*V*_D_ = 0.5 μl, *x*_H_2_O_ = 0.95) guided at 8 μm s^−1^ (see Materials and Methods and movie S5). Figure S6 shows higher speeds. All PVOH droplets contain rhodamine B for visualization. We subtracted the background from all color images. In the photographs, black triangles indicate the direction of motion. Scale bars, 1 mm.

**Fig. 6:**
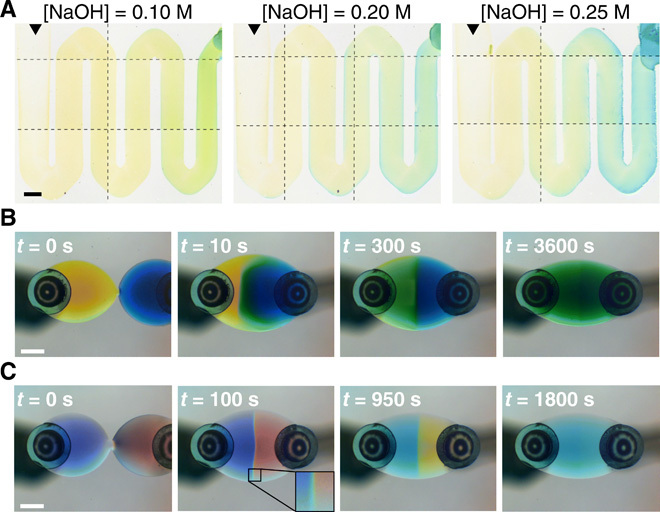
Chemical reactors with moving droplets. (**A**) Stitched photographs of color gradients in polymer deposits obtained by retracing water/PG droplets (*V*_D_ = 0.25 μl, *x*_H_2_O_ = 0.95, [PVOH] = 4 mM) containing 30 mM bromothymol blue over previous deposits by droplets (*V*_D_ = 0.15 μl, *x*_H_2_O_ = 0.95, [PVOH] = 2 mM) containing different NaOH concentrations (movie S6) (see Materials and Methods). Black triangles indicate the direction of motion. (**B** and **C**) Time sequences showing coalescence of two water/PG droplets (*V*_D_ = 0.5 μl, *x*_H_2_O_ = 0.85) controlled by two vapor sources (*R*_s_ = 640 μm) (see Materials and Methods). Droplets start coalescing at 0 s and remain visibly compartmentalized for a long time as confirmed by the flow lines in fig. S7. In (B), the droplets contain 100 mM NaOH and a dye, either methyl red (yellow, left) or bromothymol blue (blue, right); their contents mix as evidenced by the coalesced droplet turning green (movie S7). In (C), the droplets contain a pH universal indicator and either 100 mM NaOH (left) or 100 mM HCl (right); upon coalescing, an acid-base neutralization reaction occurs (inset) until the coalesced droplet turns uniform as the indicator shows qualitatively (movie S8). We subtracted the background from all images. Scale bars, 1 mm.

Next, we demonstrate the performance and versatility of our method for possible applications, ranging from printing and depositing materials to controlling reactions in space and time on pristine substrates, i.e., without requiring further modification of the underlying surface (Figs. 5 and 6). Figure 5 shows deposits from water/PG droplets containing different polymer concentrations. We guided these droplets with a large vapor source (*R*_s_ = 640 μm) placed at their leading edge to overcome the higher viscosity induced by the presence of the polymer (Materials and Methods). Figure 5A shows polymer trails left behind by moving droplets containing different concentrations of polyvinyl alcohol (PVOH), a polymer used for coating and printing applications. Visually, higher concentrations of polymer lead to thicker deposits with narrower line widths as a result of an increase in the droplet’s overall viscosity and contact angle θ_c_. The height profiles in Fig. 5B and the measurements in Fig. 5C confirm these observations quantitatively. Figure 5B also shows that typical measured thicknesses span at least two orders of magnitude with deposits as thin as ≈5 nm. For a given PVOH concentration, the resolution achievable by this printing technique in terms of average line width depends on the characteristic length of the droplet as $w\propto \sqrt[{3}]{V_{D}}$, with smaller droplets thus depositing thinner lines (Fig. 5C and fig. S5). The addition of a second degree of freedom to the in-plane displacement of the droplet allows printing arbitrary patterns in two dimensions such as sinuous lines (Fig. 5D and movie S3) and the cursive letters “ucl,” notably including double-pass portions (Fig. 5E and movie S4). Last, Fig. 5F and fig. S6 demonstrate the possibility of controlling the orientation of a polycrystalline polymer, such as polyethylene glycol (PEG) (*36*), thus leading to the formation of patterns within the deposit itself with ≈100-nm high features (fig. S6B). PEG quickly supersaturates in the deposit printed by the droplet (due to the evaporation of its more volatile component) and starts to crystallize as soon as a defect is generated (movie S5) (*37*). The resulting polycrystalline phase forms physical ridges perpendicular to a moving front whose shape depends on the droplet’s speed and determines the topography of the final pattern (fig. S6): For slower moving droplets, the front matches the droplet’s circular shape, leaving a scallop shell pattern behind, while, as the droplet speed increases, the moving front deforms into a triangular shape, which leaves a herringbone structure behind instead.

Beyond depositing material in a controlled manner (Fig. 5), our technique also allows redissolving previously deposited material within a second droplet, thus facilitating the controllable deposition of chemical reactions in space and time. For example, Fig. 6A shows the final deposits left behind by water/PG droplets containing a pH indicator (bromothymol blue) after they retrace previous deposits by basic water/PG droplets with varying concentrations of NaOH (movie S6). As a pH indicating droplet (initially yellow) retraces a previous deposit at constant speed (here *v_x_* = 25 μm s^−1^), it uptakes NaOH from the substrate, its pH increases, and its color turns from yellow to blue through green, thus printing a color gradient. We were able to regulate the steepness of this gradient, and thus the spatial variation of the printed colors, through the rate of uptake of the first deposited reactant (here NaOH), e.g., by varying its concentration in the first droplet (Fig. 6A).

Last, Fig. 6 (B and C) shows the simultaneous control of different droplets with more than one vapor source, which can be useful to trigger the mixing and the reaction of materials dissolved or suspended within them in space and time. As can be seen in Fig. 6B and movie S7, the coalescence of two droplets can be guided by controlling the separation distance between two vapor sources (see Materials and Methods). The flows in the resulting droplet are substantially different from the radially symmetric flows in a standard sessile binary droplet (*7*) and, after flowing outwards along the liquid-liquid interface between the two coalesced droplets, recirculate toward the closest source in each compartmentalized quarter (fig. S7). As a consequence, after solute exchange has occurred at the interface predominantly via diffusion, these flows promote mixing within each quarter until the coalesced droplet becomes essentially uniform in composition. Beyond mixing, we can also implement chemical reactions by the same principle. As an example, Fig. 6C and movie S8 show the acid-base neutralization reaction of NaOH and HCl: Before coalescence, the two droplets are very acidic (pH ≈ 1) and basic (pH ≈ 13), respectively; on coalescence, neutralization starts to occur at the interface where a gradient of pH forms as highlighted by the full color spectrum of the pH indicator; as the reaction proceeds, the coalesced droplet eventually reaches neutral pH uniformly.

## DISCUSSION

These examples illustrate the robustness and versatility of our method for the nonmonotonic contactless, long-range 2D manipulation of binary droplets on pristine substrates. From a fundamental side, our observation of symmetry breaking in the stable equilibrium position of the same source-droplet system highlights the intrinsic complexity of the transport mechanisms behind evaporating droplets subject to Marangoni flows. Our simplified analytical model reproduces well this symmetry breaking, and further qualitative and quantitative insight could be gained by considering the full coupling among the external vapor phase, the droplet’s composition, and the flows within it (*30*). Beyond our experimental implementation, we can expect a similar complexity to naturally emerge in the transport of droplets of any shape driven by Marangoni flows of thermal or solutal origin. In this context, the local and tunable nature of the perturbation introduced by the vapor source makes it ideal for fundamental studies of droplet motion (*21*, *25*, *38*). Because of its high sensitivity, our technique’s principle could be adapted to develop force (from nN to μN) and gas sensors. Similarly, beyond the sample applications demonstrated here, we envisage that our technique, as it does not require further substrate modification, could be used for coating applications, in printable electronics and optoelectronics, for manufacturing, as well as for the development of chemical reactors, diagnostic tools, and bioassays based on small volume liquid handling on pristine substrates (*10*, *39*–*41*).

## MATERIALS AND METHODS

### Materials

We purchased glass slides (AAAA000001##02E, Menzel Gläser) and glass capillaries (G119/02, Samco) from VWR. We purchased the largest needle used here (*R*_s_ = 350 μm) from Fisher Scientific (BD 301750) and the smaller blunt needles (*R*_s_ = 150 μm, KDS2312P; *R*_s_ = 210 μm, KDS2512P) from Farnell. We removed needles from their casing by soaking them in acetone (≥99.8%, Sigma-Aldrich). We used the following chemicals as received without further purification: PG (≥99.5%, Sigma-Aldrich), ethanol (≥99.8%, Fisher Scientific), aqueous hydrochloric acid (37%, VWR), sodium hydroxide (analytical grade, Fisher Scientific), poly(vinyl alcohol) (*M*_W_ = 9000 to 10,000 g mol^−1^, 80% hydrolyzed, Sigma-Aldrich), PEG 4000 (BDH Chemicals), rhodamine B (Acros Organics), thymol blue (Fisons), methyl red (Alfa Aesar), bromothymol blue (BDH Chemicals), and phenolphthalein (Sigma-Aldrich). We purchased a suspension of monodisperse polystyrene particles [5 μm diameter, 10 weight % (wt %)] from microParticles GmbH. We obtained deionized (DI) water (resistivity >18 megohm · cm) from a Milli-Q water purification system.

We prepared water/PG stock solutions by combining the two components in the correct mass to give the desired mole fraction (*x*_H_2_O_). For example, we made the solution at *x*_H_2_O_ = 0.95 by mixing PG (2.0 g, 26.3 mmol) with DI water (9.0 g, 500 mmol). We then used these solutions as a base for the preparation of the following stock solutions from which we made the droplets used in the experiments. We prepared a 10 mM poly(vinyl alcohol) stock solution by dissolving poly(vinyl alcohol) (95 mg) in water/PG (1 ml, *x*_H_2_O_ = 0.95). We made a 20 mM rhodamine B stock solution by dissolving rhodamine B (48 mg) in water/PG (5 ml, *x*_H_2_O_ = 0.95). We prepared a 100 mM PEG stock solution by dissolving PEG 4000 (0.20 g) in water/PG (0.5 ml, *x*_H_2_O_ = 0.95). We prepared a 2 M NaOH stock solution by dissolving NaOH (0.20 g) in water/PG (2.5 ml, *x*_H_2_O_ = 0.95). We prepared a 1 M NaOH stock solution by dissolving NaOH (0.20 g) in water/PG (5 ml, *x*_H_2_O_ = 0.85 or *x*_H_2_O_ = 0.95). We made a 1 M HCl stock solution by combining aqueous HCl (37%, 0.492 g) with PG (0.231 g) and then diluting to 5 ml with water/PG (*x*_H_2_O_ = 0.85). We made a neutral 60 mM bromothymol blue stock solution by dissolving bromothymol blue (37.5 mg) in water/PG (1 ml, *x*_H_2_O_ = 0.95) and then by neutralizing to dark yellow with a few drops of the 1 M NaOH stock solution (*x*_H_2_O_ = 0.95). We made a basic 5 mM bromothymol blue dye stock solution by dissolving bromothymol blue (6.24 mg) in a 100 mM NaOH solution (2 ml, *x*_H_2_O_ = 0.85) obtained from the 1 M stock solution (*x*_H_2_O_ = 0.85). We made a 10 mM methyl red dye solution by dissolving methyl red (2.69 mg) in a 100 mM NaOH solution (1 ml, *x*_H_2_O_ = 0.85) obtained from the 1 M stock solution (*x*_H_2_O_ = 0.85). We prepared a pH indicating stock solution, similar to Yamada’s universal indicator (*42*), by dissolving thymol blue (1.25 mg), methyl red (3.13 mg), bromothymol blue (15.0 mg), and phenolphthalein (25.0 mg) in PG (8.55 g). We added a few drops of 1 M NaOH stock solution (*x*_H_2_O_ = 0.85) to help improve the solubility of the dyes and make the solution pH neutral (green). We then added DI water (11.45 g) and diluted the solution to 25 ml using a water/PG mixture (*x*_H_2_O_ = 0.85).

### Sample preparation

We cleaned glass slides by sonicating sequentially in a 2 M NaOH ethanolic solution for 10 min, DI water for 5 min, a 1 M HCl aqueous solution for 10 min, and, finally, DI water for 5 min three times. We then dried each slide by withdrawing it from the last water bath while exposing its surface to ethanol vapor (Marangoni drying) (*19*), and removed any remaining water with a nitrogen gun. In Figs. 1 and 4 and figs. S2 and S4, we prepared binary droplets from the water/PG stock solution (*x*_H_2_O_ = 0.95) without additives. In Fig. 5 (A to E) and fig. S5, we prepared droplets from solutions of varying poly(vinyl alcohol) concentration (1 mM ≤ [PVOH] ≤ 5 mM) dyed with 10 mM rhodamine B. We made these solutions by diluting the 10 mM stock solution of poly(vinyl alcohol) with water/PG (*x*_H_2_O_ = 0.95) to 2[PVOH] and then mixing with the 20 mM rhodamine B stock solution in equal parts. In Fig. 5 (A and B), we instead prepared droplets with 6 mM poly(vinyl alcohol) and 10 mM rhodamine B by directly dissolving poly(vinyl alcohol) (28.5 mg) in 20 mM rhodamine B stock solution (2.5 ml) and water/PG (2.5 ml, *x*_H_2_O_ = 0.95). In Fig. 5F and fig. S6, we prepared droplets containing PEG directly from the 100 mM PEG stock solution. In Fig. 6A, we prepared NaOH depositing droplets ([PVOH] = 2 mM) by diluting the 2 M stock NaOH solution to 2[NaOH] with water/PG (*x*_H_2_O_ = 0.95) and then mixing with a solution of 4 mM [PVOH] in equal parts. In Fig. 6B, we prepared dye containing droplets from the stock solutions of 5 mM bromothymol blue and 10 mM methyl red. The factor of 2 between the concentrations of the dyes is to compensate for the difference in their extinction coefficients. In Fig. 6C, we prepared pH indicating droplets containing 100 mM NaOH (or 100 mM HCl) by diluting the 1 M NaOH (or 1 M HCl) stock solution (*x*_H_2_O_ = 0.85) to 200 mM with water/PG (*x*_H_2_O_ = 0.85), and then combining in equal parts with the pH indicating stock solution. In fig. S7, we prepared droplets containing microparticles for flow visualization by diluting the stock aqueous suspension of 5-μm polystyrene particles (5 μl, 10 wt %) in water/PG (995 μl, *x*_H_2_O_ = 0.85) to obtain 0.05 wt % suspensions.

### Experimental design

Unless stated otherwise, we performed each experiment by depositing a 0.5-μl binary droplet of water and PG (with or without additives) on a clean glass slide placed on a homemade inverted microscope with the possibility of switching between bright-field and dark-field illumination and equipped with a complementary metal-oxide semiconductor (CMOS) camera for monochrome (Thorlabs, DCC1545M) or color (Thorlabs, DCC1645C) imaging. A custom-made environmental chamber (Okolab) enclosed the microscope to shield the system from external air flows as well as to control temperature (*T* = 21 ± 0.5 ° C) and relative humidity (*RH* = 50 ± 5%). The entire setup was mounted on a floated optical table to reduce vibrations.

The vapor source consisted of a glass capillary (640 μm inner radius) terminated with a blunt metal needle of variable size at one end. We fixed the needle to the capillary with a silicone-based sealant. The opposite end contained 20 μl of DI water held in place through capillary forces by sealing the open end with wax. Alternatively, when a stronger vapor field was required (Figs. 5 and 6 and figs. S5 and S6), we used the same capillary on its own with DI water directly placed at its lower opening. We mounted the capillary on a three-axis micrometric stage so that we could accurately position it in space. We fixed the vertical distance of the capillary from the substrate at *z*_s_ = 0.5 mm. To determine this distance and the droplet’s contact angle θ_c_, a periscope together with a flip mirror allowed us to switch optical path so as to visualize the droplet’s side view instead of its basal plane on the CMOS cameras.

At the start of each experiment, we gently deposited the droplet under study on the clean slide with a pipette using either a disposable low-retention pipette tip (Brand, Z740080) or, for more viscous solutions (Figs. 5 and 6 and figs. S5 and S6), a standard pipette tip (Eppendorf). We carefully positioned droplets at the desired initial distance from the source of water vapor using the microscope stage, which we controlled with motorized actuators (Thorlabs, Z812B actuators with 29-nm minimum step) to guarantee the possibility of translating the droplet with respect to the source in all planar directions. We then recorded the droplet’s dynamics with low magnification with the CMOS cameras either at 1 fps (frames per second) for the droplet tracking experiment or at 10 fps elsewhere. In Fig. 4, we performed the real-time tracking of droplet motion over large distances by inputting the direct stream from the camera into a custom Matlab script, which located the droplet’s center and automatically moved the motorized stage continuously so as to maintain its relative distance *x*_s_ from the source at the initial set value. This displacement of the stage as a function of time provided the droplet’s velocity. We also performed the deposition experiments in Fig. 5 (A to E) and fig. S5 in a similar way, i.e., by moving the stage to keep the distance between droplet and source constant. We guided all droplets by holding the water vapor source at their leading edge. In Figs. 5F and 6A and fig. S6, we moved the stage in a straight line or according to a preprogrammed pattern at a constant speed. In Fig. 5F and fig. S6, we manually nucleated PEG crystallization in the supersaturated deposit with a metallic needle (movie S5). In Fig. 6 (B and C), we made two droplets coalesce by placing one droplet under a fixed vapor source (*R*_s_ = 640 μm) and a second droplet under a second identical source approximately 10 mm away, which we could move relative to the first. By reducing the intersource distance to 3.5 mm, we could make the two droplets approach gradually until coalescence.

### Estimation of droplet’s geometry and physical quantities

In our model, we obtained the spherical cap geometry of the droplet (i.e., radius *R*_D_, contact angle θ_c_, and volume *V*_D_) directly from our experimental data (fig. S2) and used it to estimate the droplet’s bulk composition (Eq. S3) and the local composition of the droplet’s free surface (Eq. 2) in time. From these composition values, we could then estimate the following physical quantities from empirical formulae: the molar volume *V*_m_ of the binary mixture in the droplet (*32*), the local value of the surface tension γ at droplet’s free surface (*31*), and its dynamic viscosity η (*32*). We obtained the value of the constant *k* = 0.15 mol Pa^−1^ m^−3^ in Eq. 2 by fitting Eq. 6 to all the experimental data on droplet’s velocity in Fig. 4. Any other aspect and equation in the model are the result of a mathematical derivation, which we have solved numerically.

### Profilometry

We measured the height profiles of the deposits in Fig. 5B and fig. S6B on the DektakXT Surface Profilometer (Bruker) with a 5-μm stylus at 5-mg force. In Fig. 5B, each profile is the average of five different profiles, which we acquired 4 μm apart using a scan resolution of 0.5 μm/point, then leveled, and smoothed them with a Hampel filter (window size: 100 data points) to remove large random spikes. We obtained the 2D profiles in fig. S6B by acquiring 1D profiles (5 μm apart in the direction perpendicular to the scan direction) with the same parameters. We leveled the data points and then smoothed them using a Gaussian-weighted moving average (window size: 30 data points along the scan direction and 4 in the perpendicular direction).

### Particle tracking velocimetry

We visualized the flows within the droplets in fig. S7 by tracking 5-μm polystyrene microparticles using a custom Matlab script. At every instant, we determined the position of these microparticles by applying a sequence of three filters to the raw images. First, we used a top-hat filter with a 3-pixel radius disc structuring element to obtain a uniform background. Then, we renormalized each pixel to the 3 pixel × 3 pixel window around it to set the centroid corresponding to each particle to a value of one. Last, we extracted particle positions by applying a binary threshold to the processed images after excluding false positives. We obtained trajectories from these data using a nearest neighbor algorithm between consecutive frames.

## Supplementary Material

aba3636_SM.pdf

aba3636_Movie_S4.mp4

aba3636_Movie_S3.mp4

aba3636_Movie_S7.mp4

aba3636_Movie_S1.mp4

aba3636_Movie_S6.mp4

aba3636_Movie_S8.mp4

aba3636_Movie_S5.mp4

aba3636_Movie_S2.mp4

## SUPPLEMENTARY MATERIALS

Supplementary material for this article is available at http://advances.sciencemag.org/cgi/content/full/6/40/eaba3636/DC1
