# Supplementary material for: Nonmonotonic contactless manipulation of binary droplets via sensing of localized vapor sources on pristine substrates
Source: Sci Adv. 2020 Sep 30;6(40):eaba3636. doi: 10.1126/sciadv.aba3636 (PMC7556999; doi:10.1126/sciadv.aba3636)
Supplement: aba3636_SM.pdf [file aba3636_SM.pdf]

Science Advances
AAAS

advances.sciencemag.org/cgi/content/full/6/40/eaba3636/DC1

Supplementary Materials for

Nonmonotonic contactless manipulation of binary droplets via sensing of localized vapor sources on pristine substrates

Robert Malinowski, Ivan P. Parkin, Giorgio Volpe*

*Corresponding author. Email: g.volpe@ucl.ac.uk

Published 30 September 2020, *Sci. Adv.* **6**, eaba3636 (2020)
DOI: 10.1126/sciadv.aba3636

The PDF file includes:

Derivations S1 to S4
Figs. S1 to S7
Legends for movies S1 to S8

Other Supplementary Material for this manuscript includes the following:

(available at advances.sciencemag.org/cgi/content/full/6/40/eaba3636/DC1)

Movies S1 to S8

Supplementary Derivation S1. Local Vapor Pressure at the Droplet's Free Surface

We can derive the steady-state solution to the diffusion equation for a flat disc source of radius R_s centered at $(x_\mathrm{s}, y_\mathrm{s}, z_\mathrm{s})$ (i.e. $(x_1, y_1, z_1) = \mathbf{0}$ in the source coordinate system) to obtain the vapor pressure of water, $p_{\mathrm{H_2O}}(r, \theta, h(r))$, at the droplet's free surface in the presence of a substrate (Fig. S2A). Specifically, for a disc source, the following differential equation is the time-dependent diffusion equation in cylindrical coordinates for the concentration $c_{\mathrm{H_2O}}(r_1, z_1, t)$ of water in the vapor phase (*27*)

$$\frac{\partial c_{\mathrm{H_2O}}}{\partial t} = D_{\mathrm{H_2O}} \left[\frac{1}{r_1} \frac{\partial}{\partial r_1} \left(r_1 \frac{\partial c_{\mathrm{H_2O}}}{\partial r_1} \right) + \frac{\partial^2 c_{\mathrm{H_2O}}}{\partial z_1^2} \right]$$

satisfying the following boundary conditions (*27*)

$$\begin{aligned} c_{\mathrm{H_2O}}(r_1, z_1, 0) &= c_{RH} \qquad && r_1 > 0, z_1 > 0, \\ c_{\mathrm{H_2O}}(r_1, 0, t) &= c_\mathrm{s} \qquad && 0 < r_1 < R_\mathrm{s}, t > 0, \\ \frac{\partial c_{\mathrm{H_2O}}}{\partial z_1} &= 0 \qquad && r_1 > R_\mathrm{s}, z_1 = 0, t > 0, \end{aligned}$$

where $D_{\mathrm{H_2O}}$ is the diffusion coefficient of water in air, c_s is the concentration of water at the source and c_{RH} is the concentration of water corresponding to the ambient relative humidity RH. Assuming steady state (*i.e.* $\frac{\partial c_{\mathrm{H_2O}}}{\partial t} = 0$) and neglecting any influence from the droplet on the diffusion of water from the source, the previous equation solves to (*27*)

$$c_{\mathrm{H_2O}} = \frac{2(c_\mathrm{s} - c_{RH})}{\pi} \sin^{-1} \left(\frac{2R_\mathrm{s}}{\sqrt{(r_1 - R_\mathrm{s})^2 + z_1^2} + \sqrt{(r_1 + R_\mathrm{s})^2 + z_1^2}} \right) + c_{RH}$$

Assuming that the vapour behaves ideally, we can convert concentration to pressure and obtain an expression for the partial pressure of water vapor along the droplet's free surface

$$P_{H_2O} = \frac{2(p_s - p_{RH})}{\pi} \sin^{-1}\left(\frac{2R_s}{\sqrt{(d-R_s)^2+(z_s-h)^2}+\sqrt{(d+R_s)^2+(z_s-h)^2}}\right) + p_{RH} \quad \text{(S1)}$$

where $d(r,\theta) = \sqrt{(x_s - r\cos\theta)^2 + (y_s - r\sin\theta)^2}$, $h(r) = \frac{(R_D^2 - r^2)}{2R_D}\theta_c$ (*29*), p_s is the saturated partial pressure of water at the source, and p_{RH} is the partial pressure of water corresponding to the ambient relative humidity RH. To account for the presence of the substrate, Eq. 1 is then obtained by adding to P_{H_2O} the vapor pressure P'_{H_2O} generated by the specular image of the disc source with respect to the surface (*28*), i.e. centered in $(x_s, y_s, -z_s)$:

$$P'_{H_2O} = \frac{2(p_s - p_{RH})}{\pi} \sin^{-1}\left(\frac{2R_s}{\sqrt{(d-R_s)^2+(z_s+h)^2}+\sqrt{(d+R_s)^2+(z_s+h)^2}}\right) \quad \text{(S2)}$$

Supplementary Derivation S2. Local Composition and Surface Tension at the Droplet's Free Surface

We can express the local water composition at the droplet's free surface as a local mole fraction $\chi_{H_2O}(r,\theta,h)$. At each step of the evaporation, we assume that, in first approximation, the evaporating droplet reaches a new value of bulk composition given by $x^{\tau}_{H_2O} = \frac{n^{\tau}_{H_2O}}{n^{\tau}_{H_2O}+n^{\tau}_{PG}}$ starting from a bulk composition $x^{b}_{H_2O}$, where $n^{\tau}_{H_2O}$ and n^{τ}_{PG} are the amounts of water and propylene glycol (in moles) in the bulk, respectively. For simplicity, we neglect intrinsic imbalances of composition (and surface tension) induced by the evaporation of the binary droplet (*24*). During evaporation, a thin liquid layer first forms near the free interface with a slightly lower content of water with respect to the initial bulk composition $x^{b}_{H_2O}$, due to the evaporation of the more volatile component (water). This thin liquid layer is of uneven composition $\chi_{H_2O}(r,\theta,h)$ along the free surface as evaporation slows down unevenly due to the presence of the source. We can therefore estimate $\chi_{H_2O}(r,\theta,h)$ using $x^{\tau}_{H_2O}$ as reference by locally enriching the new bulk

composition by an additional amount of water n'_{H_2O} so that

$$\chi_{H_2O}(r,\theta,h) = \frac{n^{\tau}_{H_2O} + n'_{H_2O}}{n^{\tau}_{H_2O} + n^{\tau}_{PG} + n'_{H_2O}}$$

For a small volume V along the droplet's free surface, as $n^{\tau}_{H_2O} = x^{\tau}_{H_2O}(n^{\tau}_{H_2O} + n^{\tau}_{PG}) = x^{\tau}_{H_2O}\frac{V}{V_m(x^{\tau}_{H_2O})}$ with $V_m(x^{\tau}_{H_2O})$ being the molar volume of the mixture at $x^{\tau}_{H_2O}$, the previous equation becomes

$$\chi_{H_2O}(r,\theta,h) = \frac{x^{\tau}_{H_2O}V_m^{-1}(x^{\tau}_{H_2O}) + \frac{n'_{H_2O}}{V}}{V_m^{-1}(x^{\tau}_{H_2O}) + \frac{n'_{H_2O}}{V}}$$

where we assumed that the addition of water n'_{H_2O} is relatively small to only cause negligible variations in total volume and molar volume. Here, we can estimate $V_m(x^{\tau}_{H_2O})$ using empirical formulae (*32*). Assuming equilibrium between condensation and vaporization dynamics at the free surface, $\frac{n'_{H_2O}}{V} = kp_{H_2O}$, i.e. the additional local concentration of water along the free surface due to the slower evaporation is proportional to the local partial pressure of water. We thus obtain Eq. 2. The constant k here expresses the balance of the rate constants associated to the condensation and vaporization of the additional amount of water along the interface when an equilibrium between the two processes is assumed. By fitting our model to all experimental data in Fig. 4, we obtain that $k = 0.15\,\mathrm{mol Pa^{-1} m^{-3}}$. Finally, given the local composition $\chi_{H_2O}(r,\theta,h)$ along the droplet's free surface, we can estimate the local surface tension $\gamma(r,\theta,h)$ using empirical formulae (*31*). Numerically, at every time step, we estimated $x^{\tau}_{H_2O}$ from the experimentally determined volume of the droplet at the following time step (see Supplementary Derivation S3).

Supplementary Derivation S3. Time Dependence of Droplet's Geometry and Composition

We estimated the geometry of the droplet over time from experimental videos of $0.5\,\mu$L droplets of initial composition $x_{H_2O} = 0.95$ evaporating in the presence of the source (Fig. S2). We measured radius R_D and contact angle θ_c directly in intervals of 25 s (Fig. S2B and C). Assuming a

spherical cap geometry (*29*), these values were then used to estimate the droplet's volume V_D as well as to define the droplet's free surface in cylindrical coordinates in time (Fig. S2A and D). We fitted each set of data to a continuous function given by a 5th-order polynomial. By assuming that no PG evaporates due to its high boiling point at 188.2° C, we can attribute all volume loss during evaporation to water, so that we can estimate the bulk composition of the droplet, to a first approximation, starting from the definition of mole fraction $x^b_{H_2O}(t) = \frac{n^b_{H_2O}(t)}{n^b_{H_2O}(t)+n^b_{PG}(0)}$ where $n^b_{H_2O}$ and n^b_{PG} are the amounts of water and propylene glycol (in moles) in the bulk, respectively. By definition,

$$x^b_{H_2O}(t) = \frac{\frac{m_{H_2O}(t)}{M_{H_2O}}}{\frac{m_{H_2O}(t)}{M_{H_2O}} + \frac{m_{PG}(0)}{M_{PG}}} = \frac{\frac{\rho_{H_2O}}{M_{H_2O}} V_{H_2O}(t)}{\frac{\rho_{H_2O}}{M_{H_2O}} V_{H_2O}(t) + \frac{\rho_{PG}}{M_{PG}} V_{PG}(0)}$$

where m_{PG}, m_{H_2O}, V_{PG}, V_{H_2O}, $M_{PG} = 76.1\,\mathrm{g\,mol^{-1}}$, $M_{H_2O} = 18.0\,\mathrm{g\,mol^{-1}}$, $\rho_{PG} = 1037\,\mathrm{kg\,m^{-3}}$ and $\rho_{H_2O} = 998\,\mathrm{kg\,m^{-3}}$ are respectively the masses, volumes, molecular weights, and densities of propylene glycol and water at 21° C. Assuming no excess volume, we can now estimate the volume of water from our experimental data (Fig. S2) as $V_{H_2O}(t) = V_D(t) - V_{PG}(0)$, so that

$$x^b_{H_2O}(t) = \frac{M_{PG}\rho_{H_2O}(V_D(t) - V^0_{PG})}{M_{PG}\rho_{H_2O}V_D(t) + (M_{H_2O}\rho_{PG} - M_{PG}\rho_{H_2O})V^0_{PG}} \quad \text{(S3)}$$

where $V^0_{PG} = 0.09\,\mu\mathrm{L}$ is the volume of propylene glycol, which we can estimate from Eq. S3 at $t = 0$ as the initial mole fraction of water is known. We then used the values of $x^b_{H_2O}$ in time to estimate the time variation of composition-dependent physical parameters, such as the equivalent mole fraction of water on the droplet's free surface (Eq. 2) and the dynamic viscosity η of the mixture (Eq. 6), so that we could evaluate the time dependence of surface tension, forces and droplet's velocity numerically.

Supplementary Derivation S4. Viscous Force on the Moving Droplet

The integral of the viscous stress (Eq. 3) over the droplet's basal area of radius R_D provides the total viscous force in the x direction as the sum of two terms, $F^v_x = F^u_x - F^\gamma_x$. Due to the circular

geometry, we can better evaluate these two terms in polar coordinates (*35*). To evaluate F_x^u, we can use the following assumptions and approximations (*35*): dissipation (i.e. the viscous force) is dominated by the contact line; for a small contact angle θ_c, $h(r) = (R_\mathrm{D} - r)\theta_\mathrm{c}$; for a droplet moving with a constant velocity v_x in the x-direction, the projection of the local velocity of the contact line along the x-direction is given by $u_x = v_x \cos^2\theta$ since the flow lines near the edge must be normal to the contact line for it to stay circular; the approximate solution to the integral $\int_0^{R_\mathrm{D}} \frac{r}{R_\mathrm{D}-r} \mathrm{d}r \approx R_\mathrm{D} \ln \frac{R_\mathrm{D}}{x_\mathrm{min}} = R_\mathrm{D}\ell_\mathrm{n}$ where we can treat the logarithm as a constant prefactor ℓ_n with x_min a microscopic cut-off of the order of the molecular size of the liquid (*35*). Typically, ℓ_n varies between 10 and 15 (*35*), and for binary droplets of water and propylene glycol of close composition and size to ours was experimentally found to be $\ell_\mathrm{n} = 11.2$ (*7*). Based on these assumptions, then

$$F_x^u = \frac{3\eta v_x}{\theta_\mathrm{c}} \int_0^{2\pi} \int_0^{R_\mathrm{D}} \frac{\cos^2\theta}{R_\mathrm{D} - r} r \mathrm{d}r \mathrm{d}\theta \approx \frac{3\pi\eta R_\mathrm{D}\ell_\mathrm{n} v_x}{\theta_\mathrm{c}}$$

where η is the dynamic viscosity of the mixture estimated at different compositions using empirical formulae (*32*). Similarly, as dissipation is dominated by the contact line (*35*), we can evaluate F_x^γ solving the following path integral along the contact line

$$F_x^\gamma = \frac{1}{2} \int_0^{2\pi} \left(R_\mathrm{D} \frac{\partial\gamma}{\partial r} \cos\theta - \frac{\partial\gamma}{\partial\theta} \sin\theta \right) R_\mathrm{D} \mathrm{d}\theta = \frac{R_\mathrm{D}}{2} \int_0^{2\pi} \left(R_\mathrm{D} \frac{\partial\gamma}{\partial r} \cos\theta - \frac{\partial\gamma}{\partial\theta} \sin\theta \right) \mathrm{d}\theta.$$

Supplementary Figures

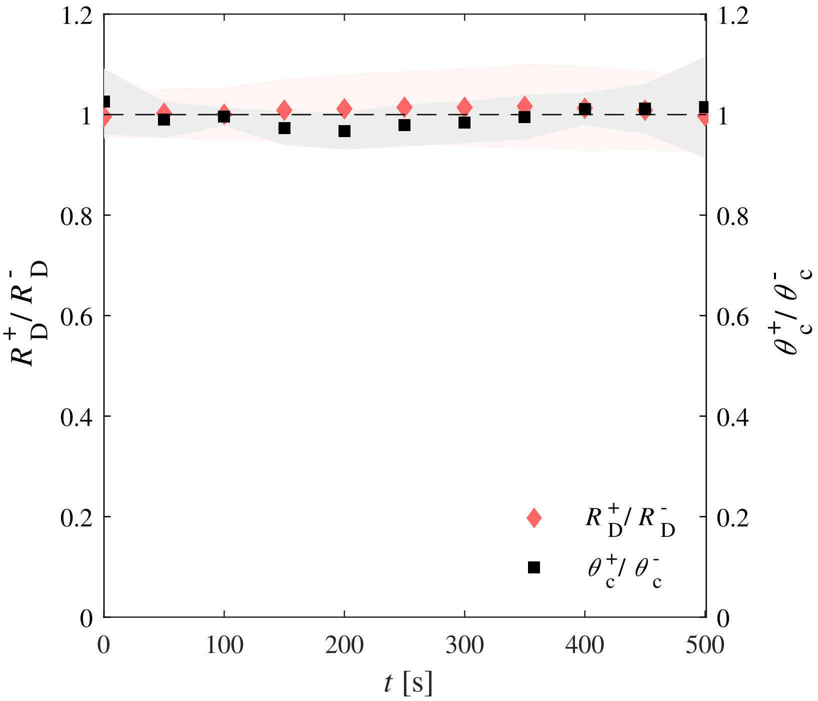

Fig. S1: **Droplet's spherical cap shape: attraction vs. repulsion.** Ratios $\frac{R_D^+}{R_D^-}$ and $\frac{\theta_c^+}{\theta_c^-}$ between the experimentally determined values of droplet's radius R_D and contact angle θ_c when attracted towards the source (R_D^+ and θ_c^+) as in Fig. 1B or repelled from it (R_D^- and θ_c^-) as in Fig. 1C ($R_s = 350\,\mu$m). Both ratios are on average close to one at all times during evaporation and show a minimal increase in droplet's radius in the case of attraction and a minimal increase in contact angle in the case of repulsion. These deviations show that the droplets are slightly extended when attracted towards the source or contracted when repelled from it in line with being either pushed towards the source or pulled away from it. These variations are however minimal and can be safely neglected in the interpretation of our experimental results. The shaded areas represent one standard deviation around the mean values calculated from at least 6 independent droplets.

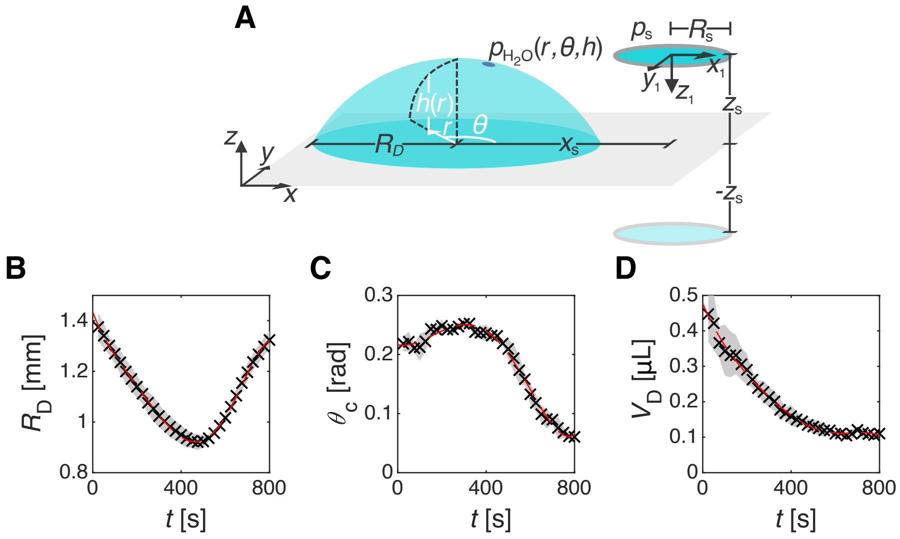

Fig. S2: **Time evolution of droplet's geometry.** (**A**) Schematic geometry of the droplet (radius R_D) and the vapor source (radius R_s, saturated vapor pressure p_s) used in the model. We define the droplet's free surface as a spherical cap in cylindrical coordinates (r, θ, h) and we associate a local water vapor pressure $p_{H_2O}(r, \theta, h)$ to each of its points (Eq. 1); x_s is the in-plane distance between droplet and source and z_s is the vertical distance between source and surface. The specular image of the source with respect to the surface is also shown at $-z_s$. The coordinate system (x_1, y_1, z_1) is centered at the source. (**B-D**) Mean experimental values (crosses) of droplet's (**B**) radius R_D, (**C**) contact angle θ_c and (**D**) volume V_D over time. The mean values are averages over 3 different droplets evaporating in the presence of the source. We estimated the droplet's volume V_D from the values of R_D and θ_c assuming a spherical cap geometry. The shaded areas represent one standard deviation around the mean values. We fitted each variable to a 5th-order polynomial (dashed line).

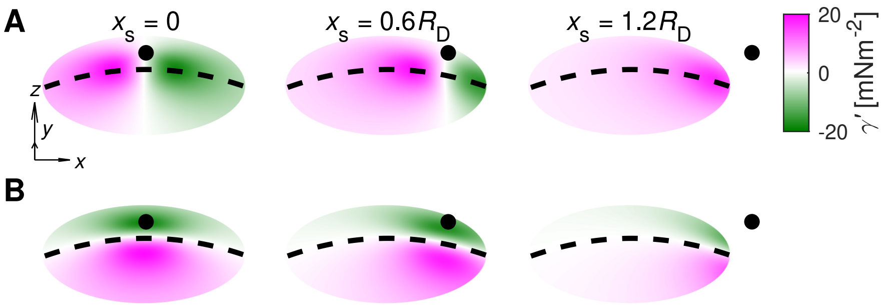

Fig. S3: **Estimated gradients of surface tension along the droplet's free surface.** (**A-B**) Estimated gradients of surface tension along (**A**) the direction of motion (x) and (**B**) the in-plane perpendicular direction (y) due to vapor sources placed at different positions x_s (black dots, $R_s = 350\,\mu$m) when evaporation starts. These gradients correspond to the profiles of surface tension shown in Fig. 2A. Dashed lines: meridians through the droplets' apices. The coordinate unit vectors correspond to 0.5 mm.

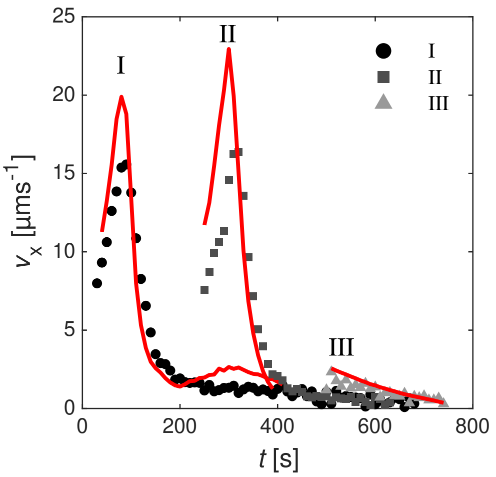

Fig. S4: **Velocity of individual droplets moving freely towards the vapor source.** Time evolution of the velocity (symbols) of three distinct droplets ($V_D = 0.5\,\mu$L, $x_{H_2O} = 0.95$) starting to move towards the vapor source ($R_s = 350\,\mu$m) from $x_s = 2$ mm (as in Fig. 1) at (I) 30 s, (II) 250 s and (III) 500 s from the beginning of their evaporation. The trajectories for the three droplets appear also in Fig. 4. The solid lines show the predicted velocity for a given droplet as interpolated from the experimental data for $R_s = 350\,\mu$m in Fig. 4. Beyond an overestimation of the peak velocity, these interpolated data predict the functional form of the droplet's velocity well.

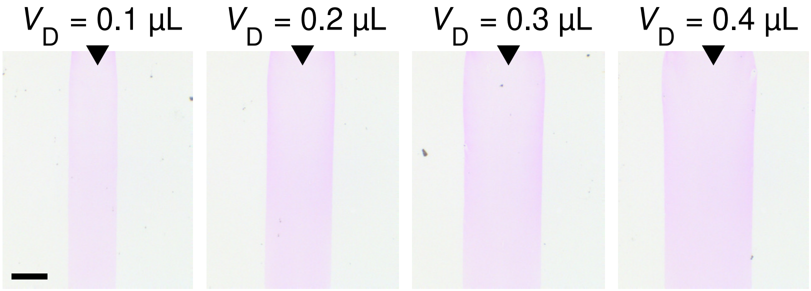

Fig. S5: **Printing with moving droplets: influence of volume at a fixed PVOH concentration.** Photographs of deposits from moving water/PG droplets (x_{H_2O} = 0.95) of same PVOH concentration ([PVOH] = 2mM) and increasing volumes. We guided all droplets along a line by holding a water vapor source (R_s = 640 μm) at their leading edge. All droplets contain rhodamine B for ease of visualization. In the photographs, we subtracted the background and the black triangles indicate the direction of motion. Scale bar: 1 mm.

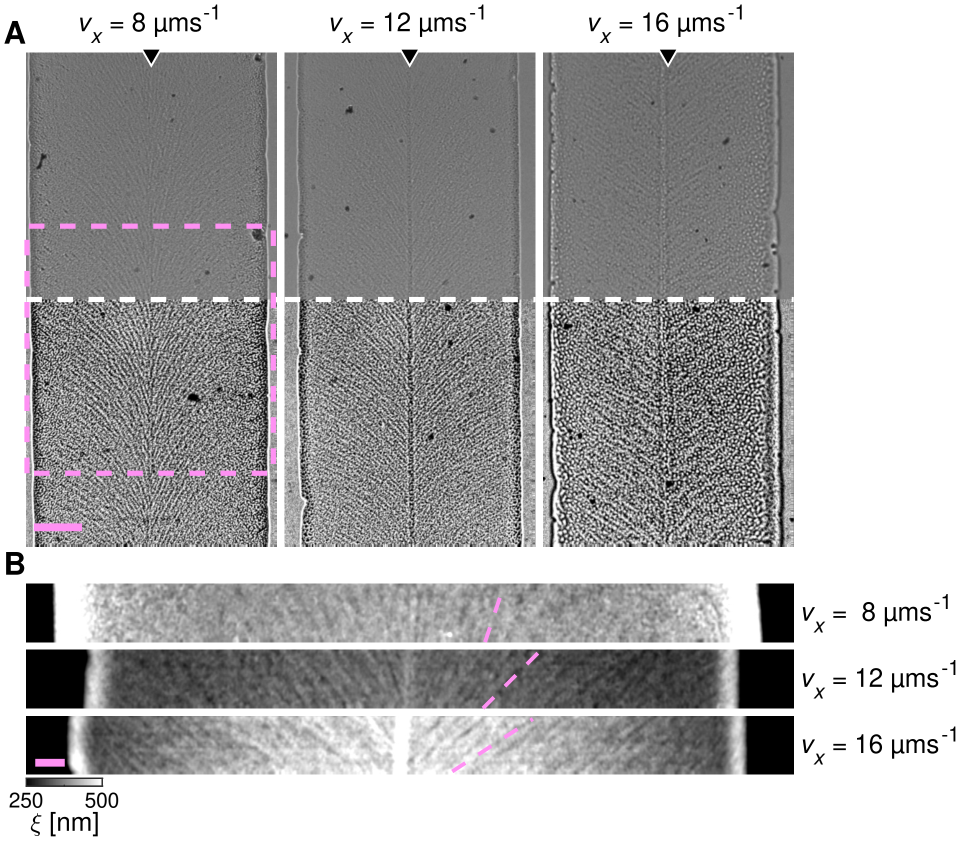

Fig. S6: **Alignment of PEG deposits with moving droplets.** (**A**) Photographs of alignment in linear polymer deposits ([PEG] $= 100\,$mM) from moving water/PG droplets ($V_D = 0.5\,\mu$L, x_{H_2O} = 0.95) guided at different speeds. The dashed lines separate two parts in the images where the top half is taken from the original photographs and the bottom half is enhanced with an edge-aware filter for contrast. The part delimited by a dashed box in the image at $8\,\mu$ms^{-1} corresponds to Fig. 5F. We guided all droplets at a constant velocity by a water vapor source ($R_s = 640\,\mu$m). The black triangles indicate the direction of motion. Scale bar: 0.5 mm. (**B**) Examples of bidimensional height maps for the polymer deposits in **A** (see Materials and Methods). The dashed lines highlight the directionality of the ridges in the deposit. Scale bar: $100\,\mu$m.

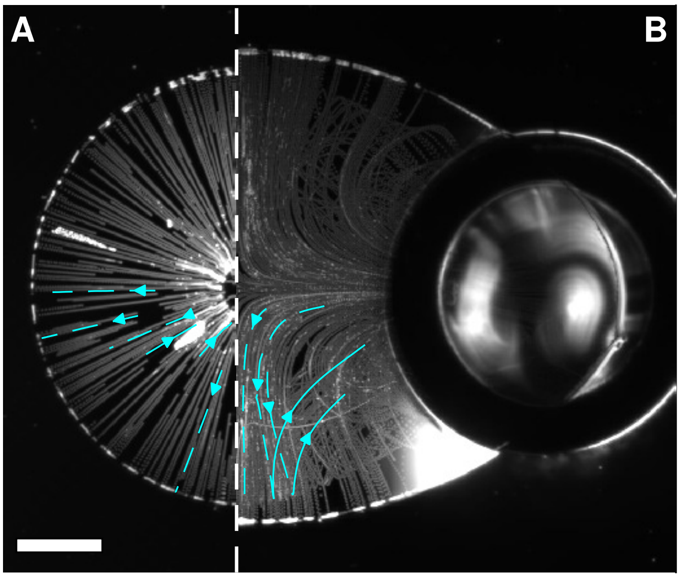

Fig. S7: **Flows within two coalesced droplets**. Flows (**A**) in a water/PG droplet ($V_D = 0.5\,\mu$L, $x_{H_2O} = 0.85$) in the absence of a water vapor source and (**B**) in a coalesced droplet formed by two similar droplets held by two vapor sources ($R_s = 640\,\mu$m) as in Figs. 6B and C (see Materials and Methods). The two cases are shown side by side and are separated by a vertical white dashed line. The cyan triangles show the directionality of typical flow lines near the droplet's basal plane (dashed lines) and along its free surface (solid lines). We measured these flows by PTV over 30 s (see Materials and Methods). Scale bar: 0.5 mm.

Supplementary Videos

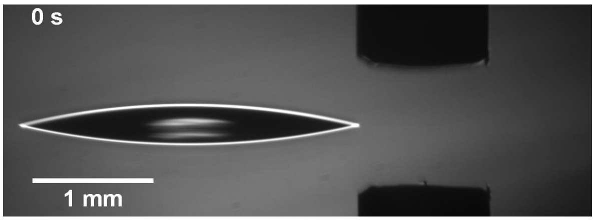

Movie S1: **Attraction of a binary droplet under an external vapor source**. Supplementary movie corresponding to the time sequence in Fig. 1B.

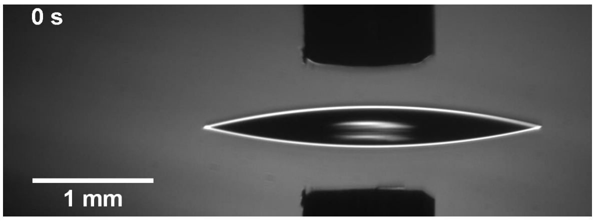

Movie S2: **Repulsion of a binary droplet under an external vapor source**. Supplementary movie corresponding to the time sequence in Fig. 1C.

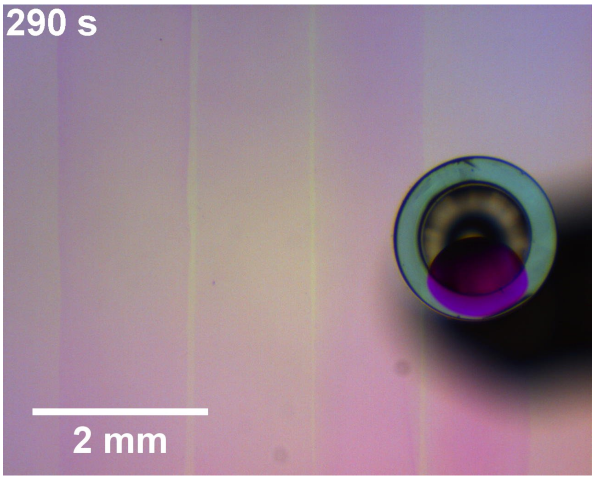

Movie S3: **Printing a serpentine with a moving droplet**. Supplementary movie corresponding to the deposit in Fig. 5D.

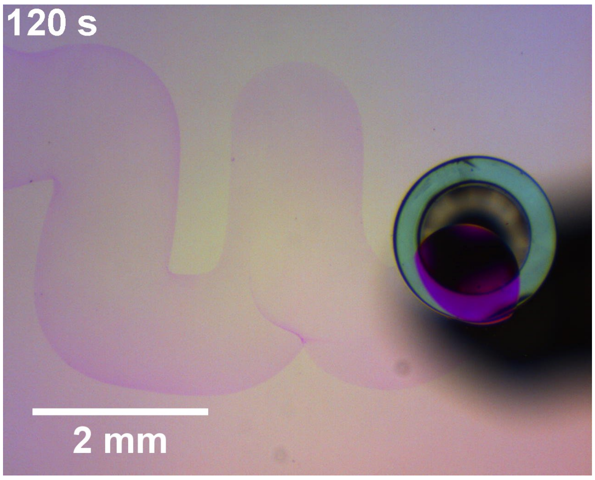

Movie S4: **Printing the letters "ucl" with a moving droplet**. Supplementary movie corresponding to the deposit in Fig. 5E.

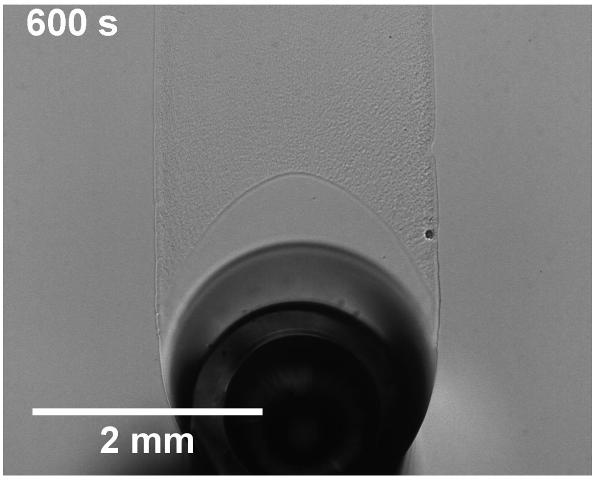

Movie S5: **Printing and aligning polymers with a moving droplet**. Supplementary movie corresponding to the deposit at $8\,\mu\mathrm{ms}^{-1}$ in Fig. 5F.

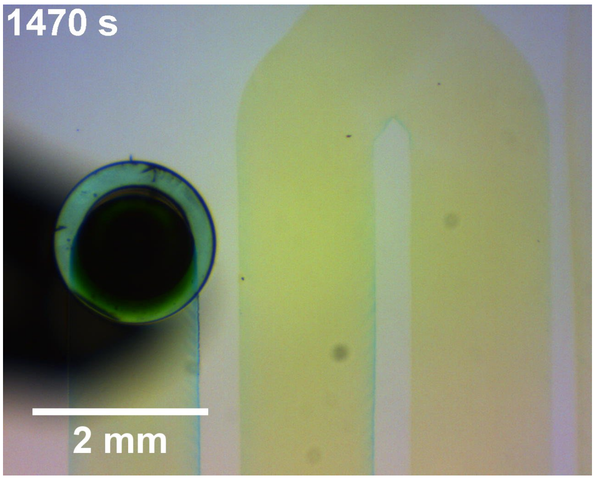

Movie S6: **Printing a reaction with moving droplets**. Supplementary movie corresponding to the deposit for $[\mathrm{NAOH}] = 0.2\,\mathrm{M}$ in Fig. 6A. The retracing of the water/PG droplet with NaOH by the water/PG droplet containing the pH indicator (bromothymol blue) starts after the first 15 seconds.

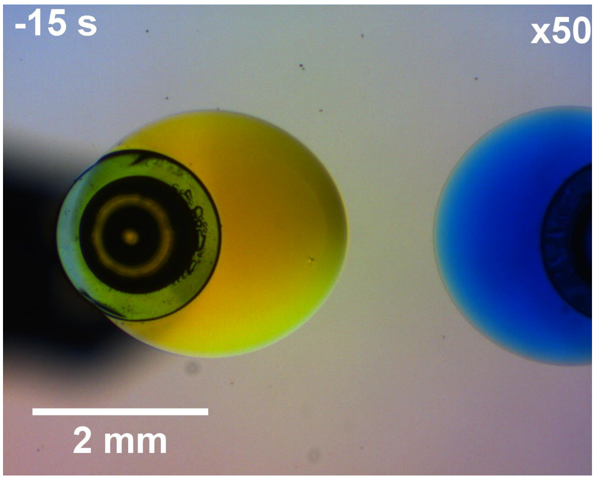

Movie S7: **Coalescing two dye-containing droplets**. Supplementary movie corresponding to the deposit in Fig. 6B. The movie is 50 times faster than real time for the first 6 seconds and 500 times faster thereafter.

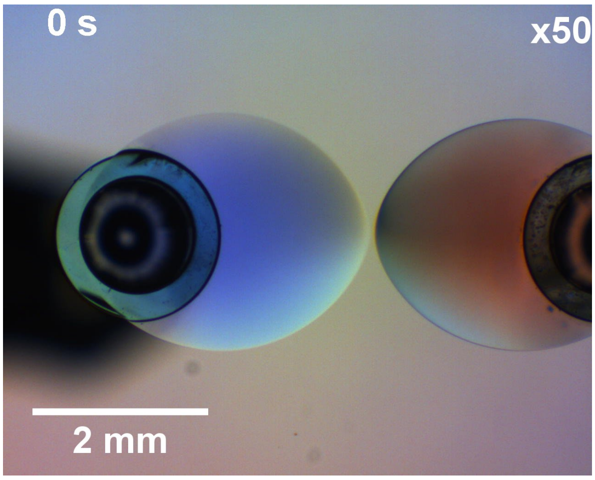

Movie S8: **Acid-base neutralization reaction in coalescing droplets**. Supplementary movie corresponding to the deposit in Fig. 6C. The movie is 50 times faster than real time for the first 6 seconds and 500 times faster thereafter.
